# Network analysis of distress, symptom burden, social support, and digital health literacy in older postoperative patients with gastric cancer

**DOI:** 10.3389/fpsyt.2026.1741661

**Published:** 2026-06-29

**Authors:** Ting Luo, Pei-rong Xu, Qi-xin Xiao, Xiao-xue Chen, Hui Zhao, Jun-sheng Peng

**Affiliations:** 1 School of Nursing, Sun Yat-sen University, Global Alliance for Chronic Care Research Center, Guangzhou, China; 2 Department of Gastric Surgery, State Key Laboratory of Oncology in South China, Guangdong Provincial Clinical Research Center for Cancer, Sun Yat-sen University Cancer Center, Guangzhou, China; 3 Department of Gastrointestinal Surgery, Department of General Surgery, Guangdong Provincial Key Laboratory of Colorectal and Pelvic Floor Diseases, The Sixth Affiliated Hospital, Sun Yat-sen University, Guangzhou, China

**Keywords:** digital health literacy, distress, gastric cancer, network analysis, psycho‐oncology, social support, symptom burden

## Abstract

**Aim:**

To use network analysis to explore the relationships among distress, symptom burden, social support, and digital health literacy in older patients with gastric cancer following surgery.

**Background:**

Digital healthcare is gaining increasing prominence and represents a promising approach to improving long-term care and psychological support for older postoperative patients with gastric cancer. However, this population frequently experiences high distress, heavy symptom burden, limited social support, and low DHL, which together constitute major barriers to the effective use of digital health resources. To date, the mechanisms underlying the interactions among these factors remain poorly understood. Therefore, this study seeks to clarify these relationships and provide empirical evidence to support the integration of digital health into geriatric oncology care.

**Methods:**

A cross-sectional study was conducted involving 767 older postoperative patients with gastric cancer at a teaching hospital between August 2024 and June 2025. Participants completed validated questionnaires, including the Brief Symptom Inventory-18 (BSI-18), the M. D. Anderson Symptom Inventory Gastrointestinal Cancer Module (MDASI-GI), the Multidimensional Scale of Perceived Social Support (MSPSS), and the eHealth Literacy Scale (e-HEALS). R software version 4.2.1 was used. Network analysis assessed the structure, centrality, stability, and accuracy of these factors.

**Results:**

Network analysis revealed that “Blue” and “Tense” of the BSI-18, and “Evaluate” of the e-HEALS were the most central nodes. “Friends” and “Sleep” acted as key bridge nodes, linking distress, symptom burden, and social support domains. The network proved stable and accurate.

**Conclusions:**

This study highlighted the item “Blue” as a central node and “Sleep” as a key bridge node. These findings suggest the potential utility of network analysis in precision nursing. Furthermore, measures such as emotional counseling for low mood, sleep optimization, and peer-navigated digital empowerment may help address the interconnected symptom pattern observed in this population.

## 1 Introduction

Gastric cancer (GC) ranks as the fifth most prevalent malignancy worldwide, with GLOBOCAN 2022 data indicating it accounted for approximately 968,000 new cases and 660,000 deaths globally (1). China bears a disproportionately high burden, contributing 37.0% of new cases and 39.5% of related deaths (2). A defining demographic feature of this disease, both globally and in China, is its predominant impact on older adults, typically those aged 60 and above (1, 2). As the global older adult population is projected to surge from 730 million in 2020 to 1.33 billion by 2040 (an 82.19% increase), the cohort of older patients with GC will rise substantially (3). This expanding patient population presents unique clinical challenges, as the quality of life (QOL) and mental health needs of older adults with cancer are frequently overlooked compared to those of younger patients (4). Surgery, as a primary treatment for GC, combined with postoperative adjuvant chemotherapy (5), can easily exacerbate the vulnerable physical and psychological state of older patients, leading to severe distress (6).

Distress is a multifactorial, unpleasant experience of psychological, social, spiritual, or physical dimensions that may impair a patient’s ability to cope effectively with cancer (7). Its clinical significance is underscored by its potential to compromise treatment adherence and QOL, prompting the National Comprehensive Cancer Network (NCCN) to designate distress as the “sixth vital sign” and recommend its systematic assessment and management (7). The prevalence of distress among older postoperative adults with GC is particularly high (79.6%), far exceeding the 43.8% observed in the general geriatric oncology population (8, 9). Because early-stage GC is often asymptomatic, many patients are diagnosed at advanced stages, which intensifies distress from the outset (10). This initial distress may be compounded during treatment (6). Of particular concern, the multiple and overlapping symptom experiences induced by treatment are critical factors that directly trigger and worsen patient suffering (11).

Defined as the cumulative physical, psychological, and social impact of illness or treatment-related symptoms (12), symptom burden serves as a critical indicator for developing personalized interventions. In older patients with GC, this burden is substantial after surgery and includes not only physical symptoms like pain and fatigue but also significant distress, such as anxiety and depression (13–15), subsequently leading to adverse outcomes such as delayed postoperative recovery and a significant decline in QOL (13, 16). Furthermore, the relationship between symptom burden and distress is commonly bidirectional: elevated physical symptoms can exacerbate distress, which in turn can amplify perceived symptom severity, creating a vicious cycle (17). This escalating experience of symptoms and distress can also undermine a patient’s ability to perceive, seek, and effectively utilize support networks (18).

Social support denotes various forms of material, informational, and emotional assistance provided by an individual’s social network, including family, friends, and colleagues (19). A prospective study has demonstrated that social support can act as a buffer against stress, enhance coping capacity and promote physical and mental health (20). As a critical protective factor, social support plays a pivotal role in healthy aging and effective cancer management. However, older patients with GC often face social vulnerabilities, such as diminishing social networks, widowhood, unemployment, and intergenerational communication barriers (21, 22). A recent study has demonstrated that low social support is a significant predictor of anxiety in older adults after gastrectomy (21). Moreover, comprehensive support programs incorporating social support components have been shown to improve surgical outcomes, reduce postoperative complications, and shorten hospital stays in these older adults (23). Therefore, understanding the role of social support within symptom-distress networks among this patient population is essential. Furthermore, in the rapidly evolving landscape of digital healthcare, identifying protective factors in disease management requires integrating traditional social support with emerging digital health technologies to collectively address complex health challenges (24).

Digital health literacy (DHL) refers to an individual’s ability to access, understand, and apply health information from electronic sources (25), and is considered by the World Health Organization to be essential for achieving health equity. While digital interventions can improve mental well-being, their effectiveness may be limited by low DHL, which is a significant barrier for older adults (26, 27). A previous study has shown that older cancer patients have substantially lower DHL scores than their younger counterparts (28), a deficit that can impede access to supportive care and intensify feelings of loneliness and anxiety (28, 29). Although previous studies in breast cancer survivors identify age (30, 31), education (28, 31), distress (28), and social support (30) as key determinants of DHL, findings in other cancer populations are inconsistent (32, 33). While the clinical importance of symptom burden, social support, and DHL is well recognized, we still understand little about how these factors actually interact during the complex postoperative recovery process. Using the individual and family self-management theory (IFSMT) as a guide (34), we frame symptom burden and social support as the context (risk and protective factors, respectively). We identify DHL as a key self-management process. It interacts with these context factors to shape psychological distress, which serves as the primary health outcome.

Traditional latent variable modeling, such as structural equation modeling, typically relies on unidirectional paths and assumes that symptoms reflect underlying constructs (15). However, this approach fails to capture the complex, bidirectional feedback loops inherent in the self-management of older patients post-gastrectomy. As seen in recent psycho-oncology work (35), network analysis (NA) does not treat distress as a latent disorder, but rather as a system of mutually reinforcing symptoms. This approach allows us to integrate clinical symptoms and psychosocial resources into a single framework (36). By doing so, we can identify specific “bridge nodes” that act as catalysts for distress or levers for intervention, providing a clearer roadmap for tailored nursing care (37).

Based on the IFSMT framework (34), this study used NA to: (1) map the symptom-resource network at the item level in older patients after gastric cancer surgery; (2) pinpoint the central nodes driving the link between physical symptoms and psychological distress; and (3) examine if DHL and social support act as bridges within this self-management system. These critical nodes serve as actionable targets for nursing interventions, ultimately enhancing self-management efficacy and improving the overall recovery trajectories of this population.

## 2 Materials and methods

### 2.1 Study design and participants

This cross-sectional study was conducted from August 2024 to June 2025. Participants were recruited from the inpatient department of a tertiary hospital in Guangzhou, China, using a convenience sampling method.

The inclusion criteria were as follows: (1) aged 60 years or older; (2) histopathologically confirmed primary GC, diagnosed in accordance with the *Chinese Society of Clinical Oncology Gastric Cancer Clinical Guidelines* (38); (3) a stable postoperative condition following radical gastrectomy, defined as having vital signs that remained stable for more than 48 hours; (4) possessing basic communication and comprehension skills in Mandarin or a local dialect; and (5) willing to participate.

The exclusion criteria were as follows: (1) a diagnosis of stage IV GC or having undergone non-curative surgery; (2) major postoperative complications requiring reoperation or intensive care; (3) a history of cognitive impairment or psychiatric disorders, including but not limited to Alzheimer’s disease, schizophrenia, and bipolar disorder; (4) the presence of other malignant tumors or serious physical illnesses, such as colorectal cancer, severe heart failure, or renal failure; or (5) concurrent enrollment in another interventional clinical trial.

### 2.2 Data analysis

We analyzed general characteristics using SPSS 25.0, presenting categorical data as frequencies/percentages and continuous data as means ± standard deviations. Network analyses were conducted in R (v4.2.1).

To ensure statistical power and minimize topological overlap, we included 18 representative items across four domains rather than full scales (39). Node selection was both data- and theory-driven. Symptom nodes (BSI-18 and MDASI-GI) were selected based on high severity and clinical prevalence. Positive resource nodes (MSPSS and e-HEALS) were chosen theoretically to reflect primary coping mechanisms. We verified node uniqueness using the *goldbricker* algorithm in the *networktools* package (39). Conceptually similar node pairs with fewer than 25% significantly different partial correlations (*α* = 0.05) were deemed redundant. Baseline distributions are provided in **Supplementary Table 7**.

Because the items were ordinal Likert-type variables, network estimation utilized a polychoric correlation matrix via *cor_auto*. We estimated a Gaussian Graphical Model (GGM) using the *bootnet* package with the EBICglasso approach (37). Setting the regularization parameter γ = 0.5, this regularization method shrinks the spurious edge weights to zero, resulting in a sparse and interpretable network. The tuning parameter was set to 0.5. Networks were visualized via *qgraph* (40). Nodes represent items, and edges signify partial correlations controlling for all other variables (41). Thicker edges indicate stronger associations. Solid lines denote positive correlations, dashed lines denote negative correlations, and missing edges imply conditional independence (42).

Core indicators were identified using standardized Z-scores for Strength and Expected Influence (via *bootnet*) (43). To evaluate practical intervenability, we calculated node predictability, representing the proportion of variance explained by neighboring nodes. Bridge Strength (via *networktools*) identified key nodes linking different subnetworks (39). Model stability and edge-weight accuracy were assessed using a non-parametric bootstrap (*n* = 2500). Centrality indices were considered stable if the correlation stability (CS) coefficient exceeded 0.50 (37). We concurrently computed 95% confidence intervals. Finally, a sensitivity analysis estimating a secondary 10-domain macro-level network confirmed the structural robustness of our primary model.

### 2.3 Sample size

The R “powerly” package (version 4.2.1) was used (44). This study incorporated 18 network nodes. Based on an empirically set sensitivity of 0.6, a statistical power of 0.80, a minimum edge weight threshold of 0.3, and a zero-order correlation matrix ranging from -0.3 to 0.6, the analysis was performed (44). To ensure network stability, the simulation recommended a minimum sample size of 765 participants. Current methodological consensus in network analysis requires large sample sizes (e.g., *N* > 10*p* or *N* ≥ 500) to ensure robust parameter estimation (45). Ultimately, 767 subjects were recruited for the study, exceeding the minimum required sample size.

### 2.4 Measurements

#### 2.4.1 General information questionnaire

The General Information Questionnaire was developed by the investigators based on a comprehensive literature review and comprised both demographic and disease-related variables. Demographic variables included sex, age, educational level, marital status, employment status, exercise habit. Clinical data encompassed chronic disease history, clinical TNM stage, surgical approaches, blood type and serum albumin.

#### 2.4.2 Measurement instruments

Four validated instruments (the Brief Symptom Inventory-18, BSI-18 (46); the M. D. Anderson Symptom Inventory Gastrointestinal Cancer Module, MDASI–GI (47); the Multidimensional Scale of Perceived Social Support, MSPSS (48); and the eHealth Literacy Scale, e-HEALS (49)) were utilized to measure distress, symptom burden, social support, and DHL, respectively. **Table 1** provides a detailed overview of these assessment tools, including their measured constructs, dimensions, scoring methods, reliability, and the specific items included in the network analysis. Although comprehensive assessments were conducted using the full scales, the network analysis specifically extracted 18 representative items across the four domains as nodes. To prevent topological overlap and ensure stable parameter estimation, item selection relied on both data- and theory-driven criteria. Specifically, we prioritized symptoms with high clinical severity and prevalence, as supported by previous studies (50–53), alongside theoretical indicators of primary coping mechanisms. **Supplementary Table 1** details the selection rationale and baseline distributions for all retained items. These instruments demonstrate satisfactory reliability, validity, and cultural applicability in Chinese cancer populations (54–57). All items are positively scored, where higher scores indicate a greater magnitude of the measured construct. The General Information Questionnaire and the full instruments are provided in **Supplementary Data 1** (**Supplementary Tables 2**-**6**).

**Table 1:** Measurement instruments summary.

Construct	Instrument	Developer (year)	All items/dimensions	Dimensions	Scoring (range)	Cronbach’s α	Items of NA
Distress	BSI-18	Derogatis et al., 2001 (46)	18/3	Depression, Anxiety, and Somatization	5-point Likert (18-90)	0.96	2, 3, 6, 7, 8, 16
Symptom burden	MDASI-GI	X. S. Wang et al., 2010 (47)	24/3	Core Items, GI Module Items, and Interference Items	11-point Likert (0-240)	0.86	1, 2, 4, 8, 14, 18
Social support	MSPSS	Zimet et al., 1988 (48)	12/3	Family, Friends, and Significant Other	7-point Likert (12-84)	0.92	2, 3, 7
Digital health Literacy	e-HEALS	Norman and Skinner 2006 (49)	8/1	Overall eHealth Literacy (single dimension)	5-point Likert (8-40)	0.98	1, 6, 8

BSI-18, the Brief Symptom Inventory-18; MDASI-GI, the M. D. Anderson Symptom Inventory Gastrointestinal Cancer Module; MSPSS, the Multidimensional Scale of Perceived Social Support; e-HEALS, the eHealth Literacy Scale; GI Module Items, Gastrointestinal Module Items.

### 2.5 Data collection and quality control

Two trained researchers from the study approached eligible participants in the inpatient department to provide a comprehensive and consistent explanation of the study’s objectives and procedures (Particular care was taken to avoid uniform responses and to correctly answer validation items). Following the signing of an electronic informed consent form, a paper questionnaire was administered to the participants. Prior to data collection, all participants were assured that their responses would be kept strictly confidential to encourage candid reporting. The entire process took approximately 25–35 minutes to complete. To ensure data integrity, a rigorous quality control process was implemented. Firstly, an assistant who was not directly involved in the study reviewed each completed questionnaire immediately for completeness and internal consistency. Secondly, an independent third-party statistician was responsible for all subsequent data entry, cleaning, and management, to minimize error and bias. Each questionnaire was marked with both issuance and return times. Those completed in less than half of the normal expected time (<15 minutes) were considered invalid and excluded from analysis.

### 2.6 Ethics statement

This study received approval from the Sun Yat-sen University School of Nursing Ethics Committee (Approval: L2024SYSU-HL-043). All procedures were conducted in strict accordance with the ethical principles outlined in the Declaration of Helsinki. The report has been prepared in adherence to the Strengthening the Reporting of Observational Studies in Epidemiology (STROBE) (58) guidelines for cross-sectional studies.

## 3 Results

**Figure 1:**
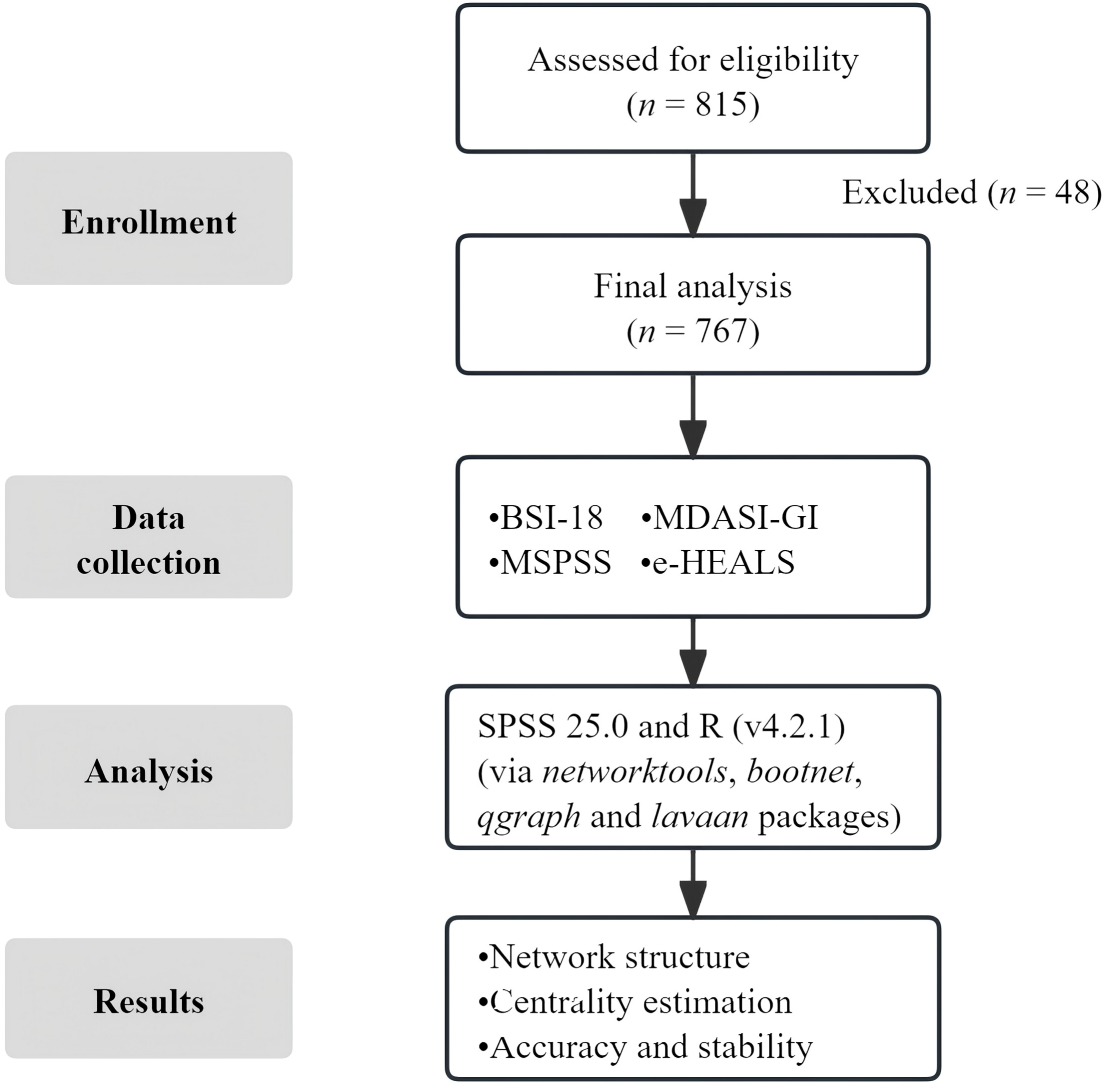
Flowchart of participant selection and data analysis procedure. BSI-18, the Brief Symptom Inventory-18; MDASI-GI, the M. D. Anderson Symptom Inventory Gastrointestinal Cancer Module; MSPSS, the Multidimensional Scale of Perceived Social Support; e-HEALS, the eHealth Literacy Scale.

This study initially assessed 815 older postoperative patients with GC. There were 48 cases not included in the final analysis, primarily due to: impaired communication capacity (*n* = 13), voluntary withdrawal (*n* = 11), invalid questionnaire responses (*n* = 8), acute health deterioration (*n* = 5), conflicting medical appointments (*n* = 5), family interference (*n* = 4), and disease progression (*n* = 2). Ultimately, a total of 767 older patients post-gastrectomy were included, and valid questionnaires were retained, yielding a response rate of 94.1%. The detailed participant flow is presented in **Figure 1**.

### 3.1 Sensitivity analysis

A sensitivity analysis using a 10-domain macro-level network demonstrated high structural and bridging consistency with the 18-item network, confirming its robustness. In the 18-item network, “Friends” (SS1) and “Sleep” (SYM1) exhibited the highest bridge strengths (2.057 and 1.768, respectively). This aligned with the domain-level network, where the “Friends” (S2) and “Core Items” (M1) domains showed the strongest conditional associations among social support, symptom burden, and distress. Additionally, the prominent bridging position of “Evaluate” (DHL2) mirrored the high bridge strength of the eHealth Literacy domain. For detailed macro-level data, please see **Supplementary Figure 1** and **Table 1**.

The goldbricker analysis revealed no topological redundancy among the 18 items. For conceptually proximate pairs, the proportions of significantly different correlations all exceeded the 0.25 threshold. Specifically, the proportion was 0.40 between “Feeling weak” (BSI-18) and “Fatigue” (MDASI-GI). For “Nausea or upset stomach” (BSI-18), the proportions compared with “Lack of appetite” (MDASI-GI) and “Feeling bloated” (MDASI-GI) were 0.47 and 0.80, respectively.

### 3.2 General participant information

Their clinical and demographic characteristics are summarized in **Table 2**. The majority were male (60.5%), with a mean age of 68.96 (SD = 6.41) years. More than half of the patients (68.2%) reported prolonged sedentary behavior, and laparoscopic surgery was the primary surgical approach (89.4%). Notably, 68.4% of patients exhibited abnormal serum albumin levels.

**Table 2:** General characteristics of older patients with GC (n = 767). .

Variable	*n*	%
Sex		
Male	464	60.5
Female	303	39.5
Age (years)		
60 - 69	476	62.1
70 - 79	239	31.1
80 - 89	52	6.8
Education level		
Primary and below	374	48.8
Junior school	274	35.7
High school or technical Secondary school	49	6.4
College or above	70	9.1
Marital status		
Married	685	89.3
Other	82	10.7
Employment status		
Employed	121	15.8
Retired	646	84.2
Exercise condition		
Sitting for long periods	523	68.2
Light activity	177	23.1
Regular exercise	67	8.7
Chronic disease history		
No	549	71.6
Yes	218	28.4
Clinical TNM stage		
I	128	16.7
II	216	28.2
III	423	55.1
Surgical approaches		
Laparoscopic surgery	686	89.4
Robotic-assisted	81	10.6
Blood type		
A	275	35.9
B	185	24.1
O	261	34.0
AB	46	6.0
Serum albumin (g/L)		
Normal	242	31.6
Abnormal	525	68.4

### 3.3 Network structure analysis

**Figure 2:**
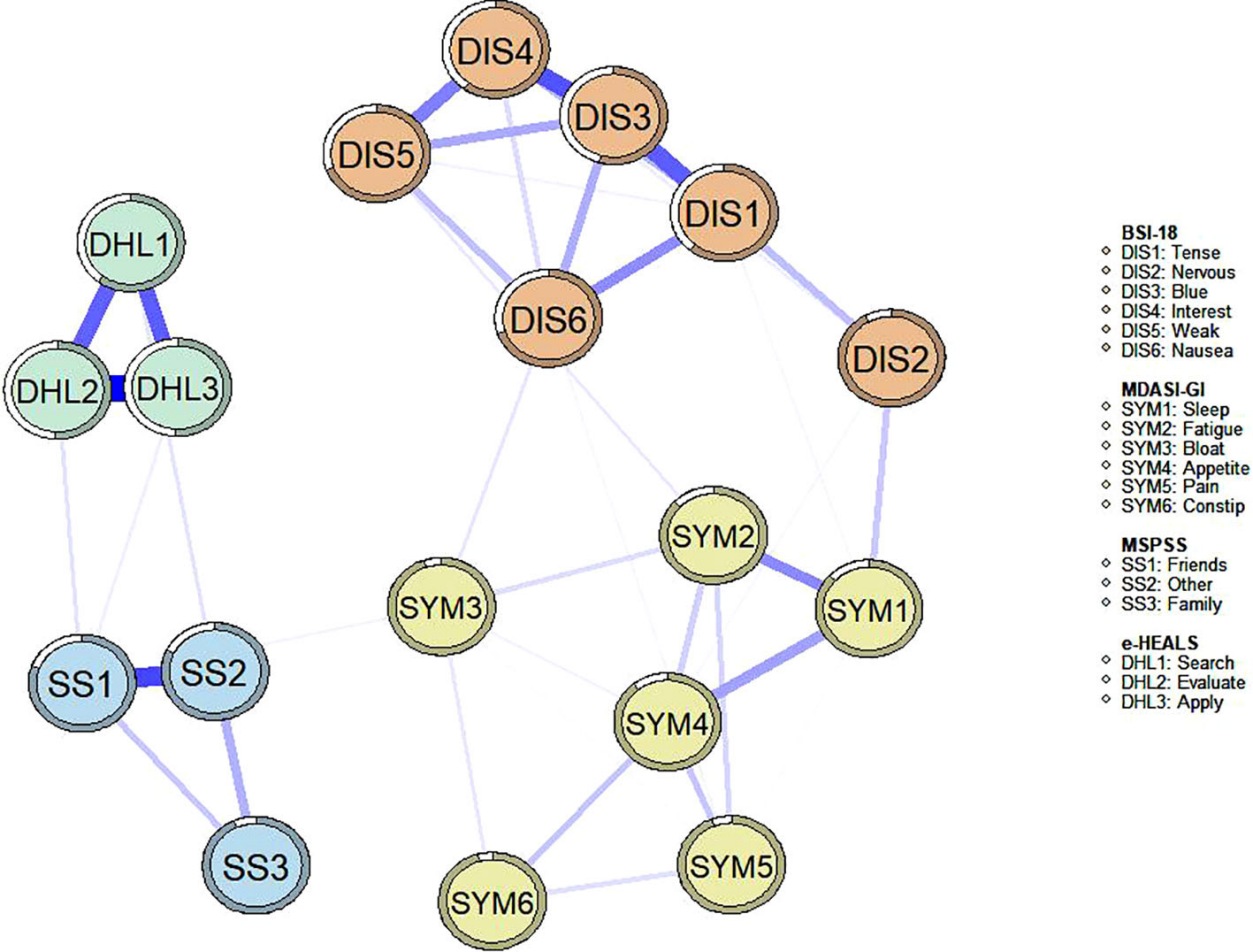
Network structure of distress, symptom burden, social support, and digital health literacy. Orange nodes represent distress (BSI-18 terms: DIS1 = “Tense”, DIS2 = “Nervous”, DIS3 = “Blue”, DIS4 = “Interest”, DIS5 = “Weak”, DIS6 = “Nausea”), green nodes represent DHL (e-HEALS terms: DHL1 = “Search”, DHL2 = “Evaluate”, DHL3 = “Apply”), yellow nodes represent symptom burden (MDASI-GI terms: SYM1 = “Sleep”, SYM2 = “Fatigue”, SYM3 = “Bloat”, SYM4 = “Appetite”, SYM5 = “Pain”, SYM6 = “Constipation”), and blue nodes represent social support (MSPSS terms: SS1 = “Friends”, SS2 = “Other”, SS3 = “Family”). BSI-18 = the Brief Symptom Inventory-18, MDASI-GI = the M. D. Anderson Symptom Inventory Gastrointestinal Cancer Module, MSPSS = the Multidimensional Scale of Perceived Social Support, e-HEALS = the eHealth Literacy Scale.

**Figure 2** presents the network structure of distress, symptom burden, social support, and DHL among older adult patients post-gastrectomy. The network consisted of 153 edges in total, of which 48 had weights greater than zero. Among all connections, the strongest edge was observed between “Evaluate” (DHL2) and “Apply” (DHL3), with an edge weight of 0.553. The next strongest edges were between “Friends” (SS1) and “Other” (SS2) (edge weight = 0.393), and between “Blue” (DIS3) and “Interest” (DIS4) (edge weight = 0.364). In addition, “Nervous” (DIS2) showed its strongest connection with “Sleep” (SYM1), with an edge weight of 0.124. For detailed data, please see the “Edge Weight Matrix” in **Supplementary Data 1**.

**Figure 3:**
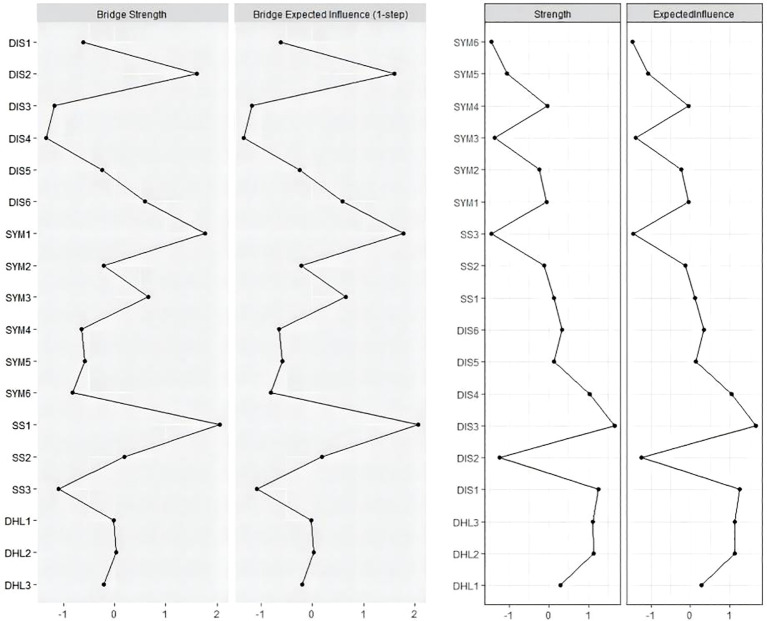
Centrality and bridge centrality indices of distress, symptom burden, social support, and digital health literacy within the network.

**Figure 4:**
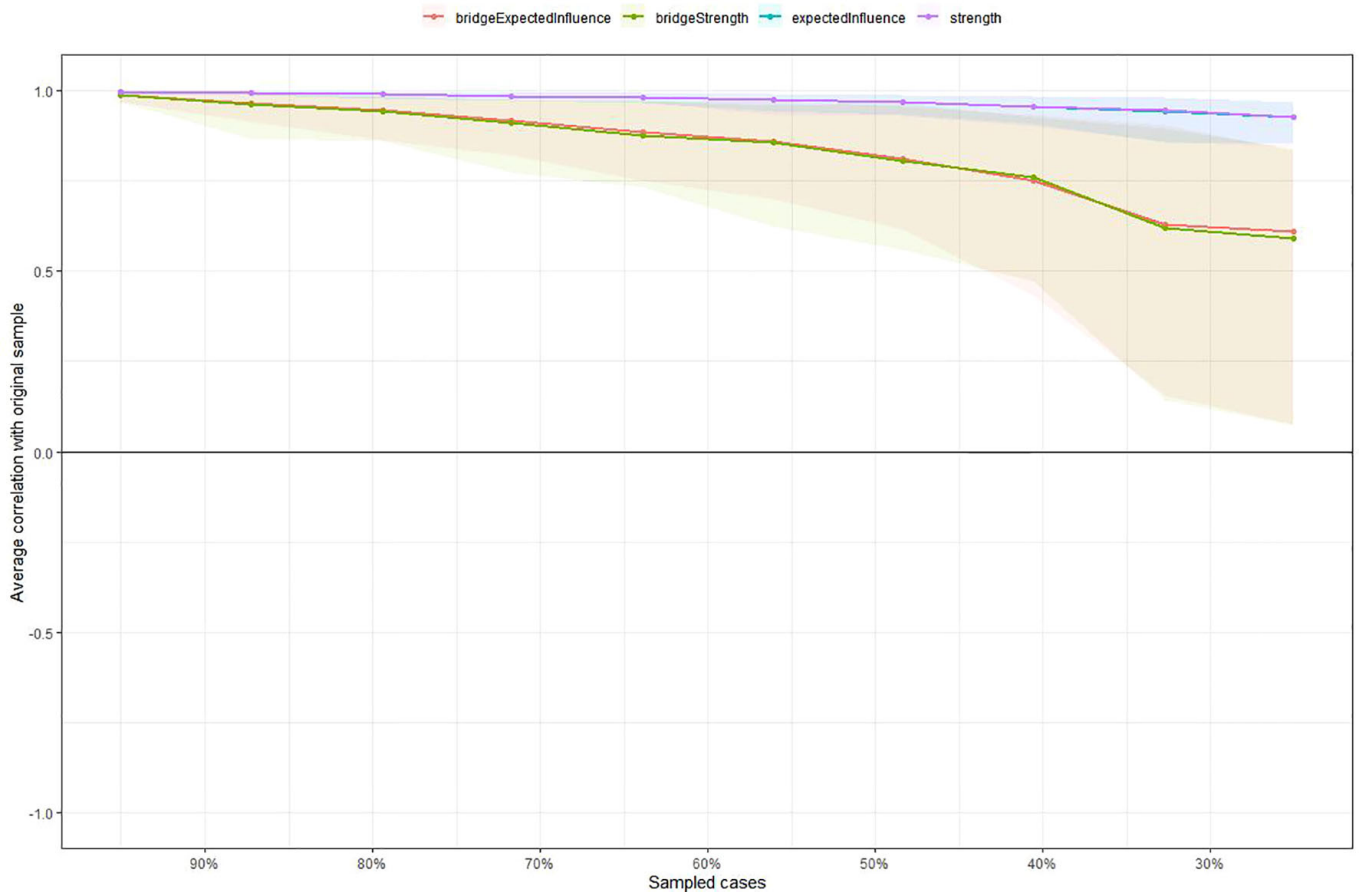
Stability of centrality indices based on case-dropping bootstrap. Average correlations between centrality indices of networks with persons dropped and the original sample. Lines indicate mean correlations, and shaded areas represent the 95% quantile range (2.5th–97.5th).

**Figure 3** displays the centrality and bridge centrality indices (S, EI, BS, BEI) and identifies the core nodes in the network. As shown in **Figure 4**, the CS-coefficient for strength centrality was relatively high; therefore, strength was adopted as the primary centrality index. Based on the integrated assessment of all centrality indices, the three most central nodes in this study were “Blue” (S = 1.660, EI = 1.660), “Tense” (S = 1.246, EI = 1.246), and “Evaluate” (S = 1.128, EI = 1.128). Additionally, “Constipation” (SYM6) demonstrated the highest predictability (0.955), indicating that a substantial proportion of its variance could be explained by its neighboring nodes.

**Table 3** presents the detailed bridge-related metrics. Among all nodes, the node representing perceived support from “Friends” (SS1) exhibited the highest BS (2.057) and corresponding BEI (2.057) across the entire network. This indicates that SS1 acts as the primary connector between the social support domain and other network communities, including symptom burden (SYM nodes) and DHL (DHL nodes). Within the symptom domain, “Sleep” (SYM1) emerged as the most impactful bridge node, with the second-highest BS (1.768) and BEI (1.768). Network topology analysis further revealed that sleep symptoms serve as a key intermediary linking the symptom domain to emotional distress (DIS nodes) and social support (SS nodes).

**Table 3:** Centrality estimates of nodes in the networks (*n* = 767).

Node ID	Item	Predictability	Strength	Expected influence	Bridge strength	Bridge expected influence
DIS1	Tense	0.609	1.246	1.246	-0.611	-0.611
DIS2	Nervous	0.918	-1.242	-1.242	1.599	1.599
DIS3	Blue	0.545	1.660	1.660	-1.177	-1.177
DIS4	Interest	0.600	1.034	1.034	-1.335	-1.335
DIS5	Weak	0.694	0.134	0.134	-0.236	-0.236
DIS6	Nausea	0.700	0.340	0.340	0.594	0.594
SYM1	Sleep	0.869	-0.056	-0.056	1.768	1.768
SYM2	Fatigue	0.870	-0.241	-0.241	-0.214	-0.214
SYM3	Bloat	0.946	-1.375	-1.375	0.660	0.660
SYM4	Appetite	0.891	-0.044	-0.044	-0.644	-0.644
SYM5	Pain	0.940	-1.068	-1.068	-0.581	-0.581
SYM6	Constipation	0.955	-1.458	-1.458	-0.809	-0.809
SS1	Friends	0.808	0.120	0.120	2.057	2.057
SS2	Other	0.820	-0.121	-0.121	0.192	0.192
SS3	Family	0.933	-1.452	-1.452	-1.082	-1.082
DHL1	Search	0.604	0.284	0.284	-0.013	-0.013
DHL2	Evaluate	0.508	1.128	1.128	0.032	0.032
DHL3	Apply	0.506	1.112	1.112	-0.200	-0.200

### 3.4 Network structure stability and accuracy tests

**Figure 5:**
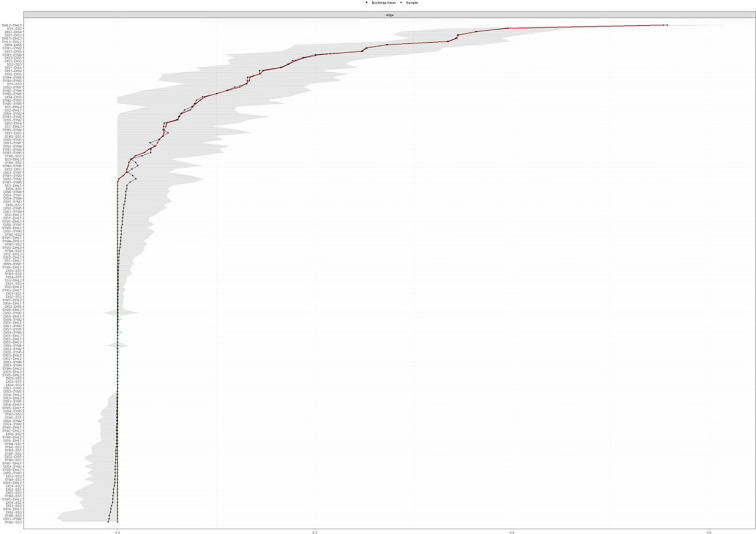
Ranking and stability assessment of network edge weights. Red lines represent the edge weights estimated from the original sample, black dots indicate the mean edge weights obtained via bootstrap, and the gray areas denote the 95% confidence intervals.

The stability evaluation of the network indicated that the stability coefficients for S and EI were both 0.750 (**Figure 4**), demonstrating acceptable network stability. Furthermore, **Figure 5** shows that the 95% confidence intervals of edge weights obtained through non-parametric bootstrap were relatively narrow, indicating good edge-weight accuracy and precision of the network estimation.

## 4 Discussion

Based on the IFSMT framework (34), this study investigates the complex relationships among distress, symptom burden, social support, and DHL in older postoperative patients with GC using NA. Among the total sample of 767 patients, 68.2% reported prolonged sedentary behavior and 68.4% exhibited abnormal albumin levels, reflecting a coexistence of impaired physiological function and nutritional risk in this population. By constructing the NA network, the present study successfully identifies specific pathways linking physical symptoms with psychological distress, suggesting potential intervention nodes for improving patients’ QOL.

The present study identifies “Blue” (DIS3, representing feeling blue) and “Tense” (DIS1, representing feeling tense or keyed up) as the core hubs within the entire network system, exhibiting strong connections with the core symptom cluster. This finding aligns with Xie et al. (59), who identified depressed mood as a core symptom in patients with colorectal cancer. Furthermore, the strong edges between psychological and physical domains support Kuang et al. (50), who highlighted the psychological vulnerability of frail older adults. For these vulnerable patients, physiological decline is associated with greater network density and higher centrality of psychological symptoms. This relationship further validates the dynamic feedback within the IFSMT framework (34). Within this specific population of older patients post-gastrectomy, psychological distress is not a single underlying condition, but rather a state associated with mutual reinforcement among physical distress symptoms (e.g., pain, nausea, and fatigue) and may be more likely to worsen in the presence of frailty. Together, the study suggests that interventions might consider targeting the strong connections between the psychological and physical domains as a potential strategy to address this interconnected state, which could have downstream benefits for rehabilitation outcomes.

The present study identifies “Friends” (SS1, perceived support from friends) and “Sleep” (SYM1, sleep disturbance) as the critical links connecting different health domains. Specifically, SS1, as an external supportive resource, shows the highest bridge strength and bridge expected influence within the overall network structure. This finding is consistent with the recent network analysis by Chirico et al. (35), which identified social support as a fundamental protective hub associated with lower emotional distress in cancer populations. Within the IFSMT theoretical framework (34), effective social resources may buffer environmental risks and support individuals’ self-management behaviors. These findings suggest that SS1 serves as the most active dynamic connector among the social support system, symptom burden, and DHL. Gu et al. (60) emphasized the critical role of internal psychological resources, such as “self-efficacy”, in breast cancer. In contrast, the present study reveals a different pattern for older patients post-gastrectomy. For this population, the connectivity of external social resources (particularly friends) appears to be more central. This peer support not only be associated with reduced perceived stress in the context of physical symptoms, but also potentially serve as an information-transfer bridge that could facilitate patients’ acceptance and use of digital health tools (21). Furthermore, within the symptom domain, SYM1 is identified as a key bridge node. According to the IFSMT framework, there is a continuous mutual association between symptoms and psychological states. As a bridge node, sleep is linked to the co-occurrence of postoperative physical discomfort and psychological distress. The bridging role of sleep is also observed by Qin et al. (61) in patients undergoing esophageal cancer surgery. Therefore, clinicians should consider sleep as more than a routine symptom; it may be a potential node to prioritize in care planning. Optimizing sleep quality could help address the interconnected relationship between physical burden and psychological distress, but this hypothesis requires future validation.

At the item level, the network analysis reveals a remarkably strong positive connection between “Evaluate” (DHL2) and “Apply” (DHL3) of health information. This finding suggests that evaluating capacity is associated with the ability to translate complex postoperative information into rehabilitation behaviors. This specific network feature in older patients with GC differs from that in prior studies. Fugmann et al. (62) indicated that in urological cancer populations, DHL was observed primarily as an independent protective factor with peripheral connections to the core symptom network in relation to psychological outcomes. However, in our current older postoperative network, DHL nodes are tightly intertwined with “Friends” support. Consistent with the IFSMT framework, this is consistent with the view that protective resources within the environmental system may support self-management. Consequently, digitally vulnerable older adults may exhibit a unique pattern that could be described as “relational digital literacy” (29). This result is in line with our hypothesis that social resources are associated with DHL. External social support, such as peer groups or mutual assistance, may help address thbe cognitive demands of independent information processing. As a result, these resources may be associated with patients’ ability to adapt to the complex postoperative rehabilitation environment (63).

## 5 Study limitations

There are several limitations. First, it employed a cross-sectional survey design, which limited the ability to establish causal relationships among distress, symptom burden, social support, and DHL. Longitudinal studies are needed to examine temporal dynamics. Second, the reliance on self-reported measures for the primary study variables (e.g., distress, symptom burden, social support, and DHL) introduces the risk of recall bias, which may affect the accuracy of data regarding past experiences or subjective states. Third, our sample exhibits clinical and demographic heterogeneity (e.g., different TNM stages, surgical approaches, etc.), but these factors were not adjusted for or stratified. This may prevent the network analysis from eliminating confounding factors; future studies should employ covariate adjustment or subgroup comparisons to analyze symptom relationships more accurately. Furthermore, this study selected representative items instead of full scales to construct the network. This approach helps avoid topological overlap and maintains stable parameter estimation. It also identifies key cross-domain bridge pathways. However, this method introduces the limitation of incomplete measurement of the domains. Consequently, the identified core nodes are specific to this sample network. Future studies should incorporate full-scale items with larger sample sizes. This would enable a more comprehensive, micro-level interaction network to be mapped.

## 6 Clinical significance

This study provides important insights into the complex links among psychological distress, symptom burden, social support, and DHL in older postoperative patients with gastric cancer. Healthcare providers should pay particular attention to older patients exhibiting high psychological vulnerability, such as feeling blue and tense. These individuals may be more likely to experience a pattern of mutually reinforcing symptoms. Practical interventions could include integrating ultra-brief screening for low mood and tension into routine assessments and implementing early preventive emotional counseling to address these core distress symptoms. For patients experiencing sleep disturbances, strict sleep hygiene and early management of pain or nausea may be helpful. These measures might help reduce the physical–psychological symptom interconnections. To reduce the potential impact of the digital divide on older adults’ self-management, clinicians could consider establishing peer-support groups. These groups may assist patients in evaluating and applying digital health information.

## 7 Conclusions

This study explored the complex relationships among psychological distress, symptom burden, social support, and DHL in older postoperative patients with gastric cancer using network analysis. The results identified the symptom of “Blue” and “Tense” as the most central symptom nodes within the estimated item-level network. Additionally, “sleep issues” and “friend support” emerged as key bridge nodes that link the physical, psychological, and social domains. The “evaluation” and “application” of digital health information were also found to be important nodes associated with translating resources into self-management behaviors. These findings suggest that interventions targeting these key nodes might help address the mutual reinforcement pattern observed among symptoms. Specifically, strategies to provide preemptive emotional counseling for low mood and tension, optimize nighttime sleep quality, and build peer-led digital support may be potentially beneficial. Such approaches could be considered to support self-management efficacy and, ultimately, may be associated with better overall recovery trajectories in these older patients.

## Data Availability

The raw data supporting the conclusions of this article will be made available by the authors, without undue reservation.
