# Supplementary material for: Network analysis of distress, symptom burden, social support, and digital health literacy in older postoperative patients with gastric cancer
Source: Front Psychiatry. 2026 Jun 29;17:1741661. doi: 10.3389/fpsyt.2026.1741661 (PMC13360191; doi:10.3389/fpsyt.2026.1741661)
Supplement: Supplementary file 4 [file Supplementaryfile1.docx]

**Supplementary Table 1.** Detailed descriptive statistics for each item of NA（*n*=767）

| **Node ID** | **Item wording** | **Original scale** | **Domain** | **Range** | **Mean** | ***SD*** | **Skewness** | **Kurtosis** | **Prevalence**  **(%)*** | **Selection rationale** |
| --- | --- | --- | --- | --- | --- | --- | --- | --- | --- | --- |
| **DIS1** | Feeling tense or keyed up | BSI-18 | anxiety | 1–5 | 1.65 | 0.88 | 1.63 | 2.81 | 45.6 | Data-driven |
| ****DIS2**** | Nervousness or shakiness inside | BSI-18 | anxiety | 1–5 | 1.42 | 0.78 | 2.40 | 6.53 | 29.3 | Data-driven |
| ****DIS3**** | Feeling blue | BSI-18 | depression | 1–5 | 1.47 | 0.79 | 2.16 | 5.38 | 33.9 | Data-driven |
| **DIS4** | Loss of interest in things | BSI-18 | depression | 1–5 | 1.31 | 0.71 | 2.77 | 8.30 | 21.3 | Data-driven |
| ****DIS5**** | Feeling weak in parts of your body | BSI-18 | somatization | 1–5 | 1.23 | 0.55 | 2.82 | 8.75 | 27.7 | Data-driven |
| ****DIS6**** | Nausea or upset stomach | BSI-18 | somatization | 1–5 | 1.78 | 0.84 | 1.45 | 3.05 | 60.0 | Data-driven |
| ****SYM1**** | Sleep disturbance | MDASI-GI | core items | 0–10 | 1.93 | 2.31 | 1.29 | 1.10 | 59.2 | Data-driven |
| ****SYM2**** | Fatigue | MDASI-GI | core items | 0–10 | 1.41 | 1.90 | 1.41 | 1.15 | 50.8 | Data-driven |
| ****SYM3**** | Feeling bloated | MDASI-GI | GI module items | 0–10 | 1.11 | 1.61 | 1.73 | 3.11 | 45.5 | Data-driven |
| ****SYM4**** | Lack of appetite | MDASI-GI | core items | 0–10 | 1.11 | 1.85 | 2.11 | 4.42 | 40.9 | Data-driven |
| **SYM5** | Pain | MDASI-GI | core items | 0–10 | 1.05 | 1.74 | 2.09 | 4.48 | 39.2 | Data-driven |
| ****SYM6**** | Constipation | MDASI-GI | GI module items | 0–10 | 0.65 | 1.46 | 2.975 | 9.89 | 26.7 | Data-driven |
| ****SS1**** | I can rely on my friends in times of difficulty | MSPSS | friends | 1–7 | 4.48 | 1.56 | -0.27 | -0.65 | N/A | Theory-driven: representing primary social support from friends. |
| **SS2** | I can share joys and sorrows with certain people (supervisor, relative, colleague) | MSPSS | significant other | 1–7 | 4.82 | 1.69 | -0.93 | 0.18 | N/A | Theory-driven: representing primary support from significant others. |
| ****SS3**** | My family provides me with concrete assistance | MSPSS | family | 1–7 | 6.29 | 0.78 | -1.16 | 1.51 | N/A | Theory-driven: representing primary social support from family. |
| **DHL1** | I know what health resources are available on the Internet | e-HEALS | literacy (single dimension) | 1–5 | 2.57 | 1.32 | 0.12 | -1.34 | N/A | Theory-driven: awareness of resources. |
| **DHI2** | I feel confident in using information from the Internet to make health decisions | e-HEALS | literacy (single dimension) | 1–5 | 2.48 | 1.28 | 0.23 | -1.14 | N/A | Theory-driven: confidence in evaluation. |
| **DHL3** | I know how to use the Internet to answer my questions about health | e-HEALS | literacy (single dimension) | 1–5 | 2.40 | 1.24 | 0.29 | -1.12 | N/A | Theory-driven: practical application. |

*SD* = standard deviation.*****Prevalence (%) : For BSI-18 items, it is defined as the percentage of patients scoring≥2; For MDASI-GI items, it is defined as the percentage of patients scoring≥1.

**Supplementary Table 2.** General Characteristics Data Questionnaire

please fit your situation“□” hit “√”or fill in the blanks in the" ".

1. Sex

□①male □②female

1. Age

(years)

1. Education level

□①Primary and below

□②Junior school

□③High school or technical Secondary school

□④College or above

1. Marital status

□①Married

□②Unmarried

□③Widowed

□④Get divorced

1. Employment status

□①Employed □②Retired

1. Exercise condition

□①Sitting for long periods

□②Light activity

□③Regular exercise

1. Chronic disease history

□①No □②Yes

1. Clinical TNM stage

□①Ⅰ

□②Ⅱ

□③Ⅲ

1. Surgical approaches

□①Laparoscopic surgery

□②Robotic-assisted

1. Blood type

□①A

□②B

□③O

□④AB

11.Serum albumin

□①Normal □②Abnormal

**Supplementary Table 3.** English Version of Brief Symptom Inventory-18 (BSI-18)

Instruction:

“Below is a list of problems people sometimes have. Please indicate how much each problem has distressed you during the past 7 days by selecting the appropriate category.”

| Item | Symptom Description | Not at all | A little bit | Moderately | Quite a bit | Extremely |
| --- | --- | --- | --- | --- | --- | --- |
| 1 | Dizziness or faintness |  |  |  |  |  |
| 2 | Loss of interest in things |  |  |  |  |  |
| 3 | Nervousness or shakiness inside |  |  |  |  |  |
| 4 | Pains in chest |  |  |  |  |  |
| 5 | Feeling lonely |  |  |  |  |  |
| 6 | Feeling tense or keyed up |  |  |  |  |  |
| 7 | Nausea or upset stomach |  |  |  |  |  |
| 8 | Feeling blue |  |  |  |  |  |
| 9 | Suddenly scared for no reason |  |  |  |  |  |
| 10 | Trouble getting your breath |  |  |  |  |  |
| 11 | Feelings of worthlessness |  |  |  |  |  |
| 12 | Spells of terror or panic |  |  |  |  |  |
| 13 | Numbness or tingling in parts of your body |  |  |  |  |  |
| 14 | Feeling hopeless about the future |  |  |  |  |  |
| 15 | Feeling so restless you couldn't sit still |  |  |  |  |  |
| 16 | Feeling weak in parts of your body |  |  |  |  |  |
| 17 | Thoughts of ending your life |  |  |  |  |  |
| 18 | Feeling fearful |  |  |  |  |  |

**Supplementary Table 4.** M. D. Anderson Symptom Inventory Gastrointestinal Module (MDASI-GI)

Part 1: Symptom Severity

“We want to know how severe your symptoms have been in the past 24 hours. For each item below, circle one number from 0 (Not present) to 10 (As bad as you can imagine) that best describes your symptom severity.”

| Item | Symptom | 0 | 1 | 2 | 3 | 4 | 5 | 6 | 7 | 8 | 9 | 10 |
| --- | --- | --- | --- | --- | --- | --- | --- | --- | --- | --- | --- | --- |
| 1 | Pain |  |  |  |  |  |  |  |  |  |  |  |
| 2 | Fatigue |  |  |  |  |  |  |  |  |  |  |  |
| 3 | Nausea |  |  |  |  |  |  |  |  |  |  |  |
| 4 | Sleep disturbance |  |  |  |  |  |  |  |  |  |  |  |
| 5 | Distress |  |  |  |  |  |  |  |  |  |  |  |
| 6 | Shortness of breath |  |  |  |  |  |  |  |  |  |  |  |
| 7 | Memory problems |  |  |  |  |  |  |  |  |  |  |  |
| 8 | Lack of appetite |  |  |  |  |  |  |  |  |  |  |  |
| 9 | Drowsiness |  |  |  |  |  |  |  |  |  |  |  |
| 10 | Dry mouth |  |  |  |  |  |  |  |  |  |  |  |
| 11 | Sadness/Depression |  |  |  |  |  |  |  |  |  |  |  |
| 12 | Vomiting |  |  |  |  |  |  |  |  |  |  |  |
| 13 | Numbness/Tingling |  |  |  |  |  |  |  |  |  |  |  |
| 14 | Constipation |  |  |  |  |  |  |  |  |  |  |  |
| 15 | Diarrhea |  |  |  |  |  |  |  |  |  |  |  |
| 16 | Difficulty swallowing |  |  |  |  |  |  |  |  |  |  |  |
| 17 | Change in taste |  |  |  |  |  |  |  |  |  |  |  |
| 18 | Feeling bloated |  |  |  |  |  |  |  |  |  |  |  |

Part 2: Symptom Interference

“Symptoms often interfere with how we feel and function. Please indicate how much your symptoms have interfered with the following activities in the past 24 hours.”

| Item | Interference Domain | 0 | 1 | 2 | 3 | 4 | 5 | 6 | 7 | 8 | 9 | 10 |
| --- | --- | --- | --- | --- | --- | --- | --- | --- | --- | --- | --- | --- |
| 19 | General activity |  |  |  |  |  |  |  |  |  |  |  |
| 20 | Mood |  |  |  |  |  |  |  |  |  |  |  |
| 21 | Work (including housework) |  |  |  |  |  |  |  |  |  |  |  |
| 22 | Relationships with others |  |  |  |  |  |  |  |  |  |  |  |
| 23 | Walking |  |  |  |  |  |  |  |  |  |  |  |
| 24 | Enjoyment of life |  |  |  |  |  |  |  |  |  |  |  |

**Supplementary Table 5. Multidimensional Scale of Perceived Social Support (MSPSS)**

Instruction: “Please indicate your level of agreement with each statement by checking the appropriate box based on your actual situation.”

| Item | Statement | Strongly Disagree | Disagree Slightly | Disagree | Neutral | Slightly Agree | Agree | Strongly Agree |
| --- | --- | --- | --- | --- | --- | --- | --- | --- |
| 1 | There is a special person (supervisor, relative, colleague) around when I am in trouble |  |  |  |  |  |  |  |
| 2 | I can share joys and sorrows with certain people (supervisor, relative, colleague) |  |  |  |  |  |  |  |
| 3 | My family provides me with concrete assistance |  |  |  |  |  |  |  |
| 4 | I get emotional help and support from my family when needed |  |  |  |  |  |  |  |
| 5 | Certain people (supervisor, relative, colleague) are real sources of comfort when I have difficulties |  |  |  |  |  |  |  |
| 6 | My friends genuinely help me |  |  |  |  |  |  |  |
| 7 | I can rely on my friends in times of difficulty |  |  |  |  |  |  |  |
| 8 | I can discuss my problems with my family |  |  |  |  |  |  |  |
| 9 | My friends share my joys and sorrows |  |  |  |  |  |  |  |
| 10 | There are people (supervisor, relative, colleague) who care about my feelings |  |  |  |  |  |  |  |
| 11 | My family willingly assists me in making decisions |  |  |  |  |  |  |  |
| 12 | I can discuss my problems with friends |  |  |  |  |  |  |  |

**Supplementary Table 6.** The eHealth Literacy Scale (e-HEALS)

Instructions: Please select the one that best fits your situation from each of the following questions

| Item | Statement | Never | Rarely | Occasionally | Frequently | Almost always |
| --- | --- | --- | --- | --- | --- | --- |
| 1 | I know what health resources are available on the Internet |  |  |  |  |  |
| 2 | I know where to find helpful health resources on the Internet |  |  |  |  |  |
| 3 | I know how to use the health information I find on the Internet to help me |  |  |  |  |  |
| 4 | I know how to find helpful health resources on the Internet |  |  |  |  |  |
| 5 | I have the skills I need to evaluate the health resources I find on the Internet |  |  |  |  |  |
| 6 | I know how to use the Internet to answer my questions about health |  |  |  |  |  |
| 7 | I can tell high-quality from low-quality health resources on the Internet |  |  |  |  |  |
| 8 | I feel confident in using information from the Internet to make health decisions |  |  |  |  |  |

**
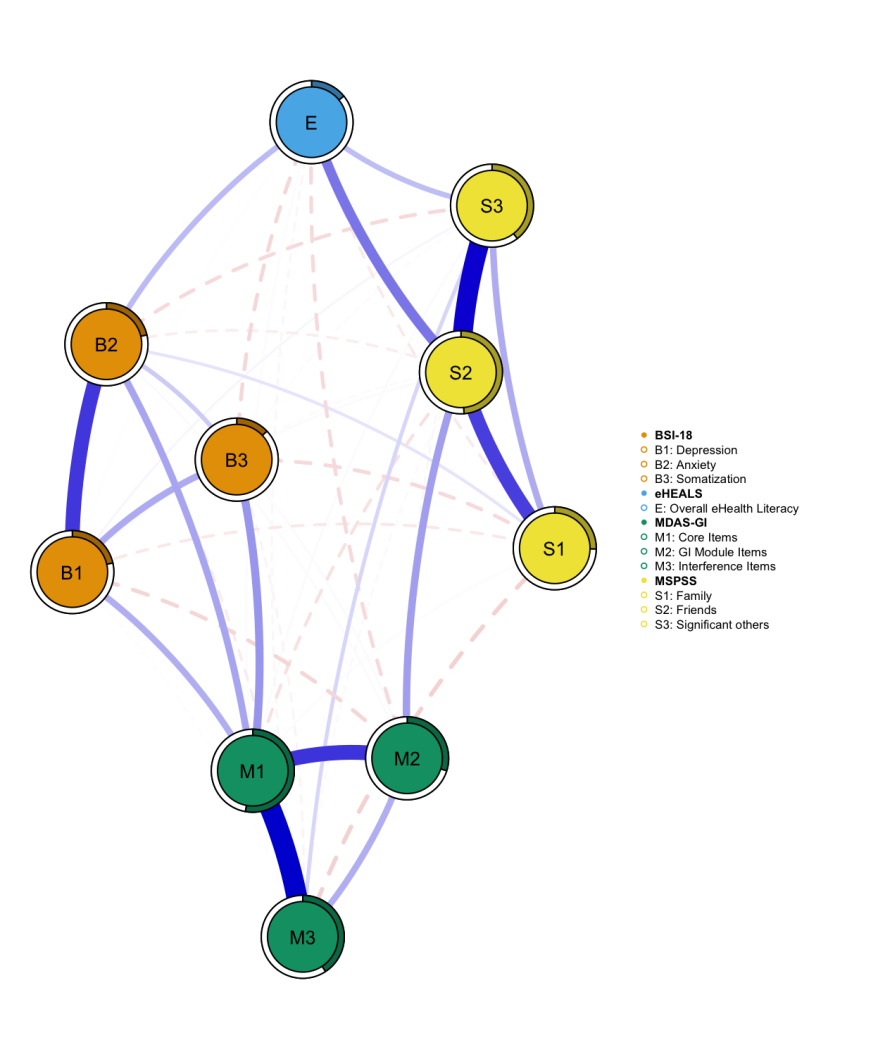
**

**Supplementary Figure 1.** Domain-level network structure.

Note: Orange nodes represent distress (BSI-18 dimensions: B1 = "Depression", B2 = "Anxiety", B3 = "Somatization"), blue nodes represent digital health literacy (e-HEALS single dimension: E = "Overall eHealth Literacy"), green nodes represent symptom burden (MDASI-GI dimensions: M1 = "Core Items", M2 = "GI Module Items", M3 = "Interference Items"), and yellow nodes represent social support (MSPSS dimensions: S1 = “Family”, S2 = “Friend”, S3 = "Significant Other"). BSI-18 = the Brief Symptom Inventory-18, MDASI-GI = the M. D. Anderson Symptom Inventory Gastrointestinal Cancer Module, MSPSS = the Multidimensional Scale of Perceived Social Support, e-HEALS = the eHealth Literacy Scale.

**Supplementary Table 7.** Centrality estimates of the domain-level network.

| **ID** | **Node** | **Predictability** | **Strength** | **Expected influence** | **Bridge strength** |
| --- | --- | --- | --- | --- | --- |
| B1 | Depression | 0.215 | 0.742 | 0.532 | 0.625 |
| B2 | Anxiety | 0.215 | 0.850 | 0.615 | 0.718 |
| B3 | Somatization | 0.130 | 0.551 | 0.242 | 0.913 |
| M1 | Core Items | 0.530 | 1.375 | 1.282 | 0.949 |
| M2 | GI Module Items | 0.299 | 0.790 | 0.514 | 0.611 |
| M3 | Interference Items | 0.407 | 0.857 | 0.556 | 0.615 |
| S1 | Family | 0.253 | 0.718 | 0.305 | 0.526 |
| S2 | Friends | 0.488 | 1.300 | 1.112 | 0.667 |
| S3 | Significant Other | 0.396 | 0.882 | 0.728 | 0.583 |
| E | Overall eHealth Literacy | 0.142 | 0.615 | 0.280 | 1.003 |
